# Cross-Modal Plasticity in Postlingual Hearing Loss Predicts Speech Perception Outcomes After Cochlear Implantation

**DOI:** 10.3390/jcm13237016

**Published:** 2024-11-21

**Authors:** Fátima Ávila-Cascajares, Clara Waleczek, Sophie Kerres, Boris Suchan, Christiane Völter

**Affiliations:** 1 Cochlear Implant Center, Department of Otorhinolaryngology, Head and Neck Surgery, Catholic Hospital Bochum, Ruhr University Bochum, Bleichstr. 15, 44787 Bochum, Germany; clara.waleczek@ruhr-uni-bochum.de (C.W.); sophiekerres@t-online.de (S.K.); 2 Clinical Neuropsychology, Faculty of Psychology, Ruhr University Bochum, Universitätsstr. 150, 44801 Bochum, Germany; boris.suchan@ruhr-uni-bochum.de; 3 International Graduate School of Neuroscience, Ruhr University Bochum, Universitätsstr. 150, 44801 Bochum, Germany; fatima.avilacascajares@ruhr-uni-bochum.de

**Keywords:** cross-modal plasticity, visual evoked potentials, postlingual hearing loss, speech perception, EEG, cochlear implantation

## Abstract

**Background:** Sensory loss may lead to intra- and cross-modal cortical reorganization. Previous research showed a significant correlation between the cross-modal contribution of the right auditory cortex to visual evoked potentials (VEP) and speech perception in cochlear implant (CI) users with prelingual hearing loss (HL), but not in those with postlingual HL. The present study aimed to explore the cortical reorganization induced by postlingual HL, particularly in the right temporal region, and how it correlates with speech perception outcome with a CI. **Material and Methods:** A total of 53 adult participants were divided into two groups according to hearing ability: 35 had normal hearing (NH) (mean age = 62.10 years (±7.48)) and 18 had profound postlingual HL (mean age = 63.78 years (±8.44)). VEPs, using a 29-channel electroencephalogram (EEG) system, were recorded preoperatively in the 18 patients scheduled for cochlear implantation and in 35 NH adults who served as the control group. Amplitudes and latencies of the P100, N100, and P200 components were analyzed across frontal, temporal, and occipital areas and compared between NH and HL subjects using repeated measures ANOVA. For the HL group, speech perception in quiet was assessed at 6 and 12 months of CI use. **Results:** No difference was found in amplitudes or latencies of the P100, N100, and P200 VEP components between the NH and HL groups. Further analysis using Spearman correlations between preoperative amplitudes and latencies of the P100, N100, and P200 VEP components at the right temporal electrode position T8 and postoperative speech perception showed that the HL group had either significantly higher or significantly lower amplitudes of the P200 component at the right temporal electrode position T8 compared to the NH controls. The HL subgroup with higher amplitudes had better speech perception than the subgroup with lower amplitudes at 6 months and 12 months of CI use. **Conclusions:** Preoperative evaluation of cortical plasticity can reveal plasticity profiles, which might help to better predict postoperative speech outcomes and adapt the rehabilitation regimen after CI activation. Further research is needed to understand the susceptibility of each component to cross-modal reorganization and their specific contribution to outcome prediction.

## 1. Introduction

Cochlear implants (CI) are neural prostheses that can restore hearing in individuals with profound hearing loss when alternative methods, such as hearing aids, are no longer helpful [1]. Unfortunately, some CI users obtain only poor speech understanding (less than 50%), and this outcome variation is not fully explained by preoperative audiometric measurements [2,3,4]. Speech perception percentages in CI users with prelingual hearing loss (HL) often correlate with activation of the auditory cortex in response to visual stimuli [5,6,7,8,9,10,11], a phenomenon known as ‘cross-modal plasticity’ [12]. It is widely suggested that cross-modal plasticity can predict speech perception outcomes in adults with postlingual HL as well [5,12,13,14,15]. However, the relationship between cross-modal plasticity and speech perception outcome is less consistent in adults with postlingual HL [5,6,13], and visual cross-modal plasticity caused by profound postlingual HL has been examined only after cochlear implantation. Importantly, understanding the neurophysiological mechanisms responsible for interindividual differences in speech perception in CI users with postlingual HL could pave the way for the development and personalization of therapeutic strategies. Therefore, in the present study, we aimed to explore preoperatively the cross-modal plasticity induced by postlingual HL, and how it correlates with speech perception outcome with a CI.

Early studies with functional magnetic resonance imaging (fMRI) in adults with congenital deafness demonstrated that visual stimulation using moving dot patterns activates the right auditory cortex [8,9,16] and that this activation is larger in subjects with profound bilateral deafness than in subjects with HL who still have residual hearing [17]. Later on, using visually evoked potentials (VEP), Buckley and Tobey [6] reported cross-modal plasticity at the right temporal area of adults with prelingual HL, but not in those with postlingual HL. In the past, many studies dealt with this topic using VEP, as EEG allows the analysis of different VEP components linked to the processing of basic stimuli features (P100) [18], to stimuli attention discrimination (N100) [19], and high-order attention allocation (P200) [20]. Unfortunately, the data available so far is inconclusive in terms of the components involved in postlingual HL and speech perception with CI. For instance, regarding adults with mild age-related hearing loss, Glick and Sharma [21] reported cross-modal plasticity in the form of reduced latencies of the three components (P100, N100 and P200) at the right temporal area, while Campbell and Sharma [22] reported larger amplitudes of the three components (P100, N100, and P200) and decreased latency of the N100 component only in the occipital area, which denotes intra-modal plasticity. In contrast, Sandmann and colleagues [18] reported increased amplitudes of the P100 component at the right temporal area in CI users compared to NH adults. Despite the different VEP components involved, intra-modal and cross-modal plasticity negatively correlated with speech perception in these studies [18,21,22]. Notably, visual cross-modal plasticity induced by HL often has right lateralization, and studies show it is correlated with speech perception in prelingual HL, mild age-related HL, and CI users with postlingual HL. However, preoperative assessment in CI candidates with postlingual HL is lacking and the components involved are not clearly identified so far.

Based on these findings, the current study aimed to (1) investigate intra- and cross-modal plasticity induced by postlingual HL by comparing the VEPs from participants with postlingual HL to those of the NH controls, and (2) to evaluate the impact of preoperative cortical reorganization on speech perception after CI provision. More specifically, we aimed to explore how the recruitment of the right temporal region to visual processing prior to CI provision is related to speech perception outcomes at 6 and 12 months of CI use. We implemented the star-circle paradigm as reported by Campbell and Sharma [22], which has been widely used to investigate cross-modal plasticity [21,22,23,24,25]. Using source localization analysis in high-density EEG, Campbell and Sharma demonstrated an activation of the medial and the superior temporal gyri during this paradigm consistent with the averaged activity recorded at a set of electrodes that overlaps with the location of electrode position T8 [21,22]. Therefore, we hypothesized that this experimental setting activity recorded at the temporal electrode positions T7 and T8 might reflect the activation of the temporal cortices, in contrast to auditory evoked potentials which are usually evaluated at the central electrode position Cz. Also, following the premise of Buckley and Tobey [6], we hypothesized that components at the temporal electrode positions should have inversed polarity when compared to the occipital positions and that polarities congruent with the occipital area reflect the contribution of the temporal area to the visual processing components and thus, to cross-modal plasticity.

## 2. Materials and Methods

The present study was approved by the Ethics Committee of the Faculty of Medicine of Ruhr University Bochum, Germany (No. 17-6197). All participants gave their written consent. This study was performed in accordance with the Declaration of Helsinki. The demographic characteristics of the 53 participants are summarized in Table 1.

### 2.1. Participants and Audiometric Testing

Adults with postlingual hearing loss (defined as onset of HL after 3 years of age) scheduled for cochlear implantation at St. Elisabeth Hospital, Bochum, were invited to participate in an EEG recording within the first week prior to surgery. Inclusion criteria were as follows: (i) the age between 45 and 80 years; (ii) severe to profound HL in the ear to be implanted (4PTA > 80 dB); and (iii) German as the native language or sufficient knowledge of German language to follow the study instructions. Exclusion criteria were as follows: (i) Uncorrected visual impairment; (ii) global cognitive impairment; and (iii) any central nervous system disease or treatment with anticholinergic medication. A total of 18 patients (mean age = 63.78 ± 8.44; 13 female) were included in the study; 6 of them presented profound HL (4PTA > 80 dB) in the contralateral ear and 12 had only severe HL (4PTA 60–80 dB) in the contralateral ear. After cochlear implantation, patients followed a standardized rehabilitation schedule of auditory speech training with a speech and language pathologist, first weekly, and later bi-weekly or monthly during a 1 to 2-year follow-up period. Speech understanding in quiet was assessed via the German Freiburg monosyllabic speech test at 65 dB before CI provision and after 6 and 12 months of CI use in the (later) implanted ear alone. Serious health problems prevented 2/18 participants from postoperative testing. Detailed individual characteristics of the HL participants are provided in Table 2. Duration of HL was assessed as the number of years between when participants no longer benefited from hearing aids and the time of EEG recording.

A group of normal-hearing participants was invited to serve as controls. Inclusion criteria were as follows: (i) age between 45 and 80 years; (ii) normal hearing (4PTA < 25 db); and (iii) German as native language or sufficient knowledge of a German language to follow the study instructions. Exclusion criteria were as follows: (i) uncorrected visual impairment; (ii) global cognitive impairment; and (iii) any central nervous system disease or treatment with anticholinergic medication. Normal hearing was verified through pure tone audiometry. Hearing thresholds were measured for pure tones of 0.25, 0.5, 0.75, 1, 1.5, 2, 3, 4, 6, and 8 kHz presented via headphones and a portable audiometer (Atmoscreen^®^, ATMOS MedizinTechnik, Lenzkirch, Germany) in a sound-isolated room. The pure tone average (4PTA) was calculated as the average of pure tone detection at 0.5, 1, 2, and 4 kHz). A total of 35 adults (mean age = 62.1 ± 7.48, 17 female) met these criteria and were included in the study as controls.

### 2.2. EEG Recording

Cortical electrical activity was recorded with 29 passive electrodes including electrode positions F7, F3, Fz, F4, F8, FC3, FCz, FC4, T7, C3, CZ, C4, T8, TP7, CP3, CPz, CP4, TP8, P3, Pz, P4, PO7, PO3, POz, PO4, PO8, O1, Oz, and O2 according to the 10/20 system (Jasper, 1958). The ground electrode was placed at the FpZ position, and linked mastoids were used as reference electrodes. Data were recorded using the BrainVision Recorder program (version 1.21.0004, Brain Products GmbH, Gilching, Germany) with a sampling rate of 500 in a quiet environment. Patients did not wear hearing aids during the EEG recording, all necessary instructions were provided in writing. All 53 participants completed the EEG appointment, in CI candidates, this took place strictly prior to cochlear implantation.

**Figure 1:**
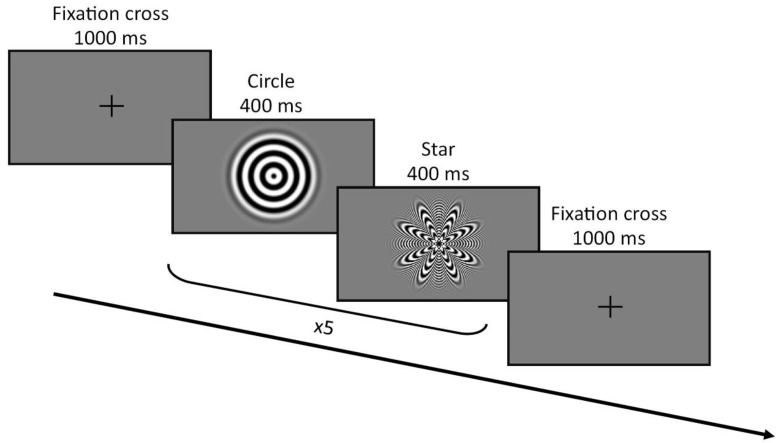
Visual evoked potentials (VEP) procedure.

Visual evoked potentials were elicited using a star-circle pattern alternating with a sinusoidal concentric pattern providing the perception of apparent motion (adapted from Campbell and Sharma [22]) implemented in Presentation^®^ (Neurobehavioral Systems Inc., Berkeley, CA, USA). Participants were comfortably seated 1 m away from a 15.6″ monitor. They received written instructions on the screen to remain still and focus on the fixation cross in the middle of the screen during the recording. Blocks of 10 trials alternating the star and circle pattern (400 ms each) were presented flanked by a 1 s fixation cross. A 2-min pause was provided after the first 15 blocks. In total, the recordings lasted 8 min, during which 300 trials (150 stars, 150 circles) were presented (Figure 1).

### 2.3. EEG Preprocessing

First, data were preprocessed using a band-pass filter with a low cutoff of 3 Hz, and high cutoff of 35 Hz, and a notch filter of 50 Hz in Brain Vision Analyzer Version 2.2. (https://www.brainproducts.com/) and independent component analysis (ICA) was used to remove artifacts caused by eye movements. Then, data were re-referenced to the common average and segmented taking 100 ms before and 600 ms after stimulus presentation. Segments were baseline corrected (using 150 ms pre-stimulus) and artifacts were automatically excluded (criterion ± 50 µV). Peak latencies and amplitudes were defined at the midpoint of the peak for each waveform component (P100, N100, and P200) in individual averages. To explore cross-modal plasticity between groups, we focused on three brain regions (frontal, temporal, and occipital) that have previously been reported to differ between adults with NH and those with HL. The electrodes used in the analysis were selected following previous studies: (i) the frontal electrodes F7, Fz, and F8 [13,23]; (ii) the temporal electrodes T7, Cz, and T8 [6,8,9,16,18,22,23,24]; and (iii) the occipital electrodes O1, Oz, and O2 [18,22,24]. The time window for peak detection was defined based on the latencies observed at Oz. In the last step, grand-average waveforms for the NH group and HL group were computed.

### 2.4. Statistical Analysis

Statistical analyses were performed using Jamovi^®^ 2.3.28 (https://www.jamovi.org). To determine group differences with respect to latency and amplitude of VEP components at electrodes of interest, repeated measures analysis of variance (rmANOVA) was performed, and significant results were further analyzed using post hoc *t*-tests with Bonferroni correction. Correlations between cortical activity and audiometric measurements before and after implantation were assessed using Spearman rank correlation. Homogeneity of variance was examined with Levene’s test and normality of distribution was assessed using the Shapiro–Wilk test. In the absence of a sphericity assumption, Greenhouse–Geisser correction was used. In cases of violation of assumptions of homogeneity or normality, non-parametric statistics were used.

## 3. Results

### 3.1. Evaluation of the Visual Evoked Potentials

**Figure 2:**
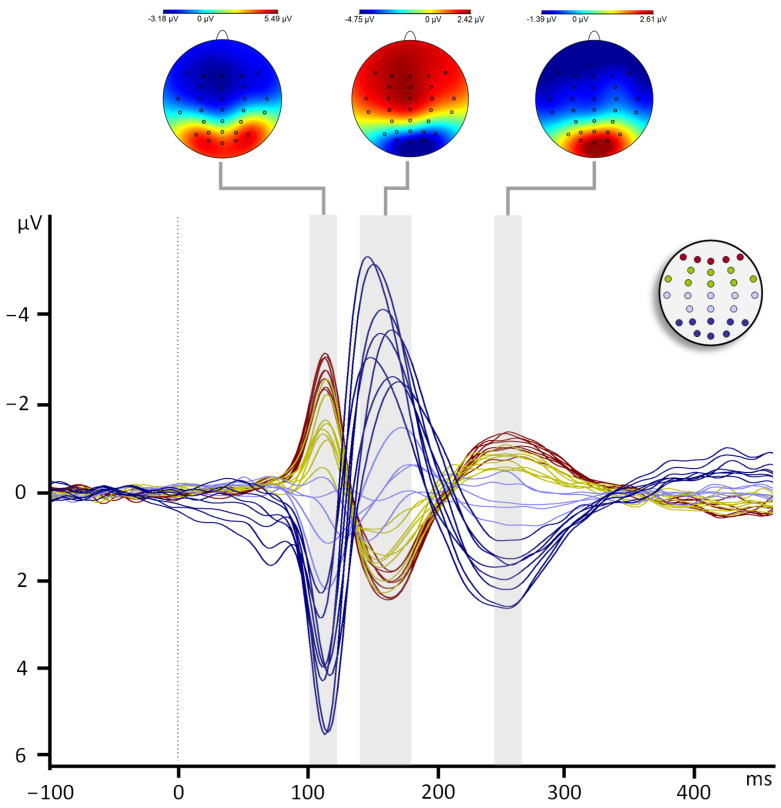
Grand averages across the 29 channels. The mapping view (top) and detection time window (shaded area) are shown for each component. Red signals positive voltages and blue signals negative voltages. Grand averages for all channels are superposed and color-coded depending on their antero-posterior location, as shown in the top right of the schema. Dark red: frontal channels, green: central and temporal channels, lilac: parietal channels, dark blue: parieto-occipital and occipital channels.

**Figure 3:**
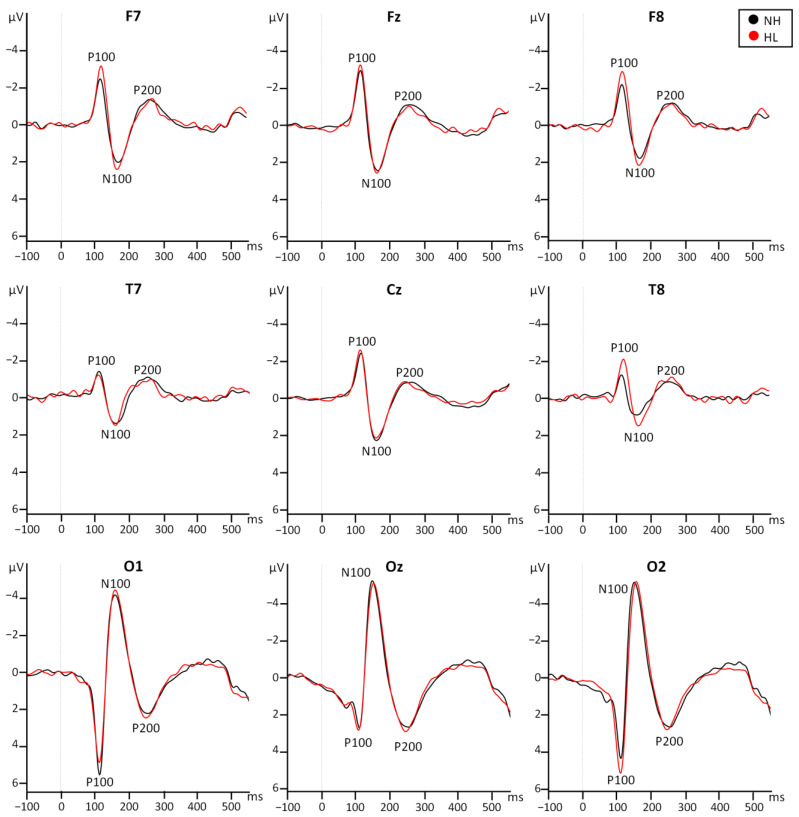
Grand averages at the frontal (**top row**), temporal (**middle row**), and occipital (**bottom row**) electrode positions for the NH group (black) and the HL group (red).

Main components P100, N100, and P200 were observed at all electrodes of interest, with the largest amplitudes at occipital and parieto-occipital electrode positions. Consistently with previous reports [23,26,27] the polarity of the VEP components was inverted after the midline because the data were re-referenced to the common average (Figure 2). Therefore, in frontal, central, and temporal electrodes P100 and P200 have negative amplitudes and N100 has positive amplitudes (Figure 3). Amplitudes and latencies of each component were extracted from the frontal, temporal, and occipital electrodes. Repeated measures ANOVA (Appendix A, Table A1) were performed including the factors Group (NH and HL), Row (frontal, temporal, and occipital), and Laterality (left, middle, right). No main effect for the factor group was observed for the amplitude or latency of any of the three components (*p* > 0.05). The significant effects reported below confirm the distribution across the scalp of the VEP components.

*Amplitude of the P100 component.* The interaction Row*Laterality was significant (F(2.53,129.28) = 4.62, *p* = 0.007), with smaller amplitudes of the P100 component found at Oz compared to those at O1 (t(51) = 4.81, *p* < 0.001) and at O2 (t(51) = −4.23, *p* = 0.004). There was a significant triple interaction Row*Laterality*Group (F(2.53,129.28) = 3.96, *p* = 0.014); however, no post hoc comparison was significant (*p* > 0.05).

*Amplitude of the N100 component.* A significant interaction Row*Laterality (F(2.79,142.42) = 5.46, *p* = 0.002) was found. Amplitudes of the N100 component were larger at the occipital row compared to the frontal (t(51) = 13.97, *p* < 0.001) and temporal (t(51) = 13.36, *p* < 0.001) rows, and larger at the frontal than the temporal row (t(51) = 5.72, *p* < 0.001). Amplitudes of the N100 component were smaller at the right compared to left (t(51) = 3.28, *p* = 0.006) and middle (t(51) = 3.24, *p* = 0.006) electrode positions. Further analysis of the Row*Laterality interaction showed smaller amplitudes at F8 compared to Fz (t(51) = 3.55, *p* = 0.03) and smaller at O1 than Oz (t(51) = 4.53, *p* = 0.001).

*Amplitude of the P200 component.* Significant main effects for the factors Row (F(1.09,56.05) = 196.69, *p* < 0.001) and Laterality (F(1.70,87.04) = 12.61, *p* < 0.001) were observed. Amplitudes of the P200 component were larger at the occipital compared to the frontal (t(51) = −14.13, *p* < 0.001) and the temporal (t(51) = −14.41, *p* < 0.001) rows, and larger at the frontal compared to the temporal row (t(51) = −2.89, *p* = 0.017). Amplitudes were larger in the middle than the left (t(51) = −4.89, *p* < 0.001) and right (t(51) = 2.65, *p* = 0.032) electrode positions, and smaller at left than right electrode positions (t(51) = −2.52, *p* = 0.045).

*Latency of the P100 component.* The Row*Laterality interaction was significant (F(3.39,172.92) = 4.21, *p* = 0.005). Latency was shorter in Oz than O1 (t(51) = 3.85, *p* = 0.012).

*Latency of the N100 component.* There was a significant Row*Laterality interaction (F(3.44,175.49) = 4.48, *p* = 0.003). Latency at the frontal row was longer than at the temporal (t(51) = 3.63, p = 0.002) and occipital (t(51) = 5.16, *p* < 0.001) rows.

*Latency of the P200 component.* There were no significant main effects of the factor Row (F(1.84,93.95 = 2.02, *p* = 0.142), the factor Laterality (F(1.95,99.58) = 3.11, *p* = 0.05), or any interaction. A full account of the latencies and amplitudes can be found in Appendix A, Table A2.

### 3.2. Preoperative Audiometric Factors and Postoperative Speech Perception (HL Group)

Speech perception scores at 6 and 12 months of CI use did not correlate with age or hearing loss in the contralateral ear. Moderate correlations were observed between the duration of HL and speech perception at 6 months of CI use, and between the severity of hearing loss in the implanted ear and speech perception at 12 months of CI use. Results are summarized in Table 3.

### 3.3. VEP at Electrode Position T8 and Postoperative Speech Perception Correlations

Based on previous findings, we analyzed the correlation between preoperative activity of the right temporal area and postoperative speech perception. Since current studies in adults with postlingual HL have not been consistent in terms of the components involved, we had no hypothesis regarding specific components.

**Figure 4:**
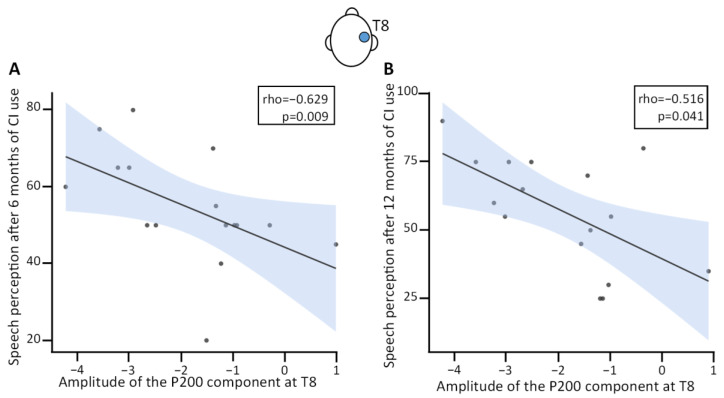
Correlation between amplitude of the P200 component at T8 and speech perception (% correct) at (**A**) 6 months and (**B**) 12 months of cochlear implant use. CI, cochlear implant. The shaded area denotes the standard deviation.

Spearman correlations were calculated between the amplitudes of the three VEP components (P100, N100, and P200) at electrode position T8 and speech perception measured by the Freiburg speech test at 65 dB after 6 and 12 months of CI use (Table 4). Data from one participant was excluded from this analysis because amplitudes of the N100 and P200 components at electrode position T8 were statistical outliers. Therefore, all further calculations were carried out with a sample size of 15. Given the relatively small sample size, we call for caution in generalizing these results beyond our dataset. No significant correlation was found between postoperative speech perception and the amplitudes and latencies of the P100 component or the latency of the N100 component. Speech perception at 6 months of CI use significantly correlated with the latency (rho(13) = −0.54, *p* = 0.038) and amplitude of the P200 component at electrode position T8 (rho(13) = −0.594, *p* = 0.019; Figure 4A). Moderate correlations were observed for the amplitude of the N100 component at electrode position T8 and speech perception at 6 and 12 months of CI use, and between the amplitude of the P200 component at electrode position T8 and speech perception at 12 months of CI use (Figure 4B).

**Figure 5:**
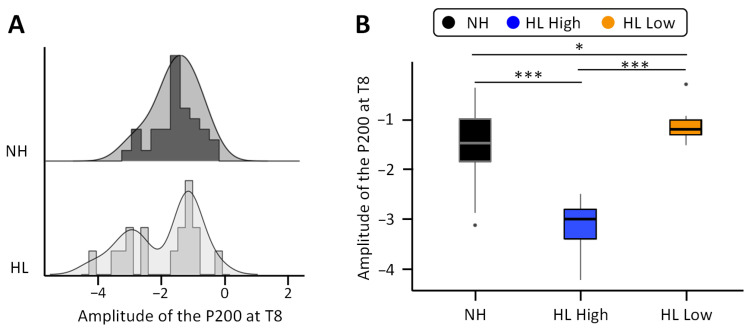
(**A**) Group distributions of the amplitude of the P200 component at T8 in the normal hearing group (**top**, black) and the hearing loss group (HL) (**bottom**, light gray). (**B**) Amplitude of the P200 component at T8 in the normal hearing group (black) and the two subgroups of with hearing loss (high in blue and low in orange). Note. * *p* < 0.05, *** *p* < 0.001.

Given that the amplitude of the P200 component at T8 showed the strongest correlation with speech perception at 6 months of CI use, we decided to analyze interindividual differences for this component back in the full sample (excluding the outlier). While in the NH group the amplitude of the P200 component at T8 showed a normal distribution centered around −1.52 µV, the amplitude distribution in the HL group extended beyond the lower bound (−1.752) of the 95% confidence interval for the mean of the NH group (Figure 5A). To further explore the effect of increased amplitudes, we split the participants with HL according to the amplitude of the P200 component at T8 with a cut-off threshold of −2 µV into two subgroups of CI candidates: one with significantly larger amplitudes (high, *n* = 7, t(40) = 5.88, *p* < 0.001, Cohen’s d = 2.44) and one with significantly smaller amplitudes (low, *n* = 10, Welch’s t(31.16) = −2.63, *p* = 0.013, Cohen’s d = −0.76) than the NH controls (Figure 5B) according to independent *t*-tests. The amplitude for both HL subgroups was also significantly different from each other (t(15) = −9.12, *p* < 0.001, Cohen’s d = −4.49).

Next, we compared the two HL subgroups in terms of age, severity of hearing loss in the implanted and the contralateral ear, and duration of hearing loss. We did not find statistically significant differences. However, according to the effect sizes (Cohen’s d) the tendency to larger age and longer duration of hearing loss in the low group compared to the high group should not be overlooked (see Table 5).

**Figure 6:**
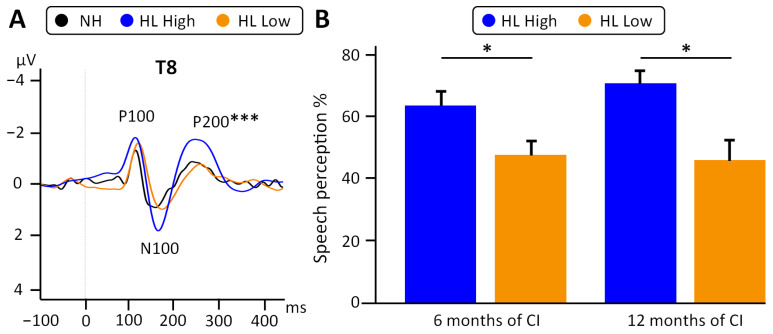
(**A**) Grand averages at T8 of the normal hearing controls (black line), “HL high” (blue) and “HL low” (orange). One-way ANOVA with factor Group (NH, HL high, and HL low) was significant (F(2,14) = 27.92, *p* < 0.001). (**B**) Speech perception after 6 and 12 of CI use (6MoPostOP, 12MoPostOP, respectively) was significantly better in the “HL high” (blue) than the “HL low” group (orange). * *p* < 0.05, *** *p* < 0.001.

In terms of postoperative speech perception, consistent with the Spearman correlations, participants in the high subgroup reached at least 50% speech perception (mean = 63.6 ± 11.4) at 6 months of CI use and up to 90% at 12 months of use (mean = 70.7 ± 11.7). In contrast, in the low subgroup, only a few patients reached a speech perception of >50% at 6 months (*n* = 2/8, mean = 48.1 ± 14.1) or at 12 months of CI use (*n* = 3/8; mean = 47.5 ± 20.5). There were statistically significant differences in speech perception outcomes between both groups at 6 months (t(13) = 2.30, *p* = 0.038, Cohen’s d = 1.19) and 12 months of CI use (t(13) = 2.63, *p* = 0.021, Cohen’s d = 1.36, Figure 6).

## 4. Discussion

Cross-modal plasticity has frequently been posited as a means to distinguish good from poor CI performers [24] and to predict postoperative speech outcomes [15,18]. The most common interpretation—which can only be proven by preoperative EEG analysis—is that poor speech performance with a CI is caused by deprivation-induced cross-modal plasticity. However, most studies were performed months or even years after CI provision [12,18,24,28] and describe a combined effect of HL and hearing restoration by the CI. In this study, we addressed (1) whether potentials evoked by visual stimulation differ between NH adults and adults with profound postlingual HL before cochlear implantation, (2) whether visual stimulation recruits the right temporal area of adults with postlingual HL, and if so, (3) how does this cross-modal plasticity relate to postoperative speech perception.

### 4.1. Cortical Reorganization Induced by Hearing Loss

Previous literature on VEP differences between adults with NH and those with mild-moderate postlingual HL [21,22,23] have shown a large degree of variability in results both in terms of the components involved and the brain areas undergoing cortical reorganization [21,22,23]. In the present study, we found no difference either in amplitudes or latencies of the VEP components P100, N100, and P200 between the NH and HL groups. One explanation could be that cortical plasticity is larger at the early stages of hearing loss and it stabilizes over time. For instance, resting-state positron emission tomography (PET) studies have found that the temporal cortices become hypoactive shortly after the onset of HL and gradually increase up to normal levels as the duration of HL increases [13,29]. Another explanation could be that the variability within the hearing-impaired group precludes the comparison with the NH controls. For instance, an in-depth analysis of our results revealed that the amplitude of the P200 component at the right temporal electrode position T8 increased in a subset of the HL group compared to the NH. Differences within cohorts with postlingual HL have been reported previously with fRMI during reading-based phonological tasks [14,30]. The persistent variability within the patients with postlingual HL makes it necessary to study differences within this group, as we discuss in the following section.

### 4.2. VEP Response in the Right Temporal Area and Postoperative Speech Perception

Based on abundant reports of cross-modal plasticity in the right temporal region of individuals with prelingual HL [6,8,9,11,16], mild-moderate age-related HL [22,23], and CI-users [18], we decided to investigate the correlation between the amplitudes and latencies of the three main VEP components: P100, N100, and P200 at the right temporal electrode position T8 and postoperative speech perception.

In contrast to the study of Sandmann and colleagues [18], we encountered no evidence of cross-modal reorganization of the P100 component. They found smaller amplitudes at occipital electrode positions and larger amplitudes at the right temporal cortex in CI users compared to individuals with NH, and the latter negatively correlated to speech perception. Given that the aforementioned study also examined differences between luminance ratios, it is possible that condition and group effects in the P100 component depend on basic stimuli features.

We observed a non-significant, but moderate tendency to higher speech perception scores as amplitudes of the N100 component at T8 became larger. This lack of correlation has been previously reported in CI users with postlingual HL, in contrast to CI users with prelingual HL [6]. In general, CI users with postlingual HL appear to be more resistant to cross-modal plasticity than those with prelingual HL [13]. The use of hearing aids might reduce cross-modal reorganization, as described in ref. [21]. Their VEP study showed that cross-modal plasticity found in subjects with untreated mild-moderate HL reverted to NH values after a 6-month intervention with hearing aids. However, our sample size could limit the statistical power to detect differences in this component and the role of this component still needs to be explored in larger cohorts.

Preoperatively, we found shorter latencies and larger amplitudes of the P200 component at T8 in patients that reach better speech perception outcomes 6 and 12 months after cochlear implantation. Shorter latencies and larger amplitudes are considered typical markers of cross-modal reorganization [23,24,31,32]. Previously, larger amplitudes of the P200 component at occipital electrode positions have been reported in good performers [24] and similar results have been observed in adults with early mild HL [22]. While different regions were involved in the present study, we interpret the increased amplitudes of the P200 component in the “high” subgroup as a correlate of enhanced attention to the sensory stimuli that will be useful for speech perception after CI provision.

As mentioned in the introduction, there is a large variability among studies in terms of the components involved in cross-modal plasticity relevant to speech perception with a cochlear implant. Some relevant sources of variability are the onset of severe hearing loss (prelingual vs. postlingual) [6] and the timepoint of cross-modal plasticity evaluation (preoperative [5,15,33] vs. postoperative [18,24,28,31]). Given the relatively small sample size in this and in previous studies we do not intend to establish the P200 component as the key marker of postoperative speech prediction, but we seek to raise awareness of the necessity for preoperative measurements of cross-modal plasticity in larger cohorts and their potential to predict and to explain postoperative outcomes.

### 4.3. Divergent Modulation in Deprivation-Induced Cross-Modal Plasticity

As expected [23], polarities of the VEP components were inverted over the midline, such that the P200 component had negative amplitudes in the temporal areas. Some participants with HL in the present study preserved the NH-like polarity of the P200 component but exhibited larger amplitudes than NH (“high amplitude subgroup”) and showed better postoperative speech perception than their peers with reversed polarity and smaller amplitudes of the P200 component at electrode position T8 (“low amplitude subgroup”). To explain the different audiometric outcomes of postoperative speech perception after CI use Lazard et al. proposed different trajectories or profiles of plasticity associated with outcome [34]. We suggest that our “high” and “low” subgroups might fit their “awakening” and “stabilization” profiles, described to be associated with high and low benefits from CI use, respectively. This might be explained by the theoretical background provided by Kral and Sharma [35], which posits that the excitability of the auditory cortex is upregulated as an adaptive response to the reduced auditory input in HL, and that cross-modal responsiveness arises from the already existing heteromodal connections, which can change from modulatory to driving forces in the absence of auditory input [35]. In the present study, increased amplitudes of the P200 component at electrode position T8 (“high subgroup”) were consistent with increased adaptive excitability based on functional preoperative connections that might support speech perception after CI provision. On the contrary, polarities more aligned to the visual cortex might result from reinforced connections between the visual and auditory cortex, such that the visual stimuli not only modulate but also drive responses in the auditory cortex. We propose that cross-modal plasticity depends on the number and strength of heteromodal connections prior to HL. This divergence in plasticity patterns might (1) account for the lack of differences between NH and HL in the present study, and (2) contribute to discrepancies across studies, depending on the proportion of study participants with a given plasticity profile.

### 4.4. Cross-Modal Plasticity as a Predictive Tool in Hearing Rehabilitation

Speech outcome after cochlear implantation largely varies across individuals and the reasons are not yet fully understood [3,4]. The close interaction between the visual and auditory cortices has been suggested as a preoperative marker of the postoperative outcome in CI recipients [36]. Clinical multifactorial models used so far to predict postoperative outcomes account for approximately 22% of the variability in speech perception outcomes [37]. While there has been progress in CI outcome prediction in recent decades, most models rely on various factors such as the duration of HL or maximum preoperative speech perception [38]. While the duration of HL is frequently hard to determine in people with gradual postlingual HL [15,33], preoperative speech perception can only be measured in patients with residual hearing, and the outcome in patients with total HL cannot be approximated by this parameter. In contrast, preoperative VEP measurements do not rely on medical records or patients’ memory and can provide an objective and up-to-date report of deprivation-induced plasticity and availability of the temporal cortex for sensory restoration. In our study, there was a moderate correlation between the duration of hearing loss and speech perception outcome at 6 months of CI use, as well as a tendency for a larger duration of hearing loss in the subgroup with low amplitudes of the P200 component at electrode position T8, who had lower postoperative speech perception outcomes. This tendency to better speech perception outcomes in patients with shorter duration of hearing loss is consistent with previous literature [39] and should be taken into account in future studies on cross-modal plasticity. Another factor that should not be overlooked is age. Even though age did not significantly correlate in the present study with postoperative speech perception, there was a moderate tendency to older ages in the low group compared to the high group. This opens two relevant possibilities that could be further explored experimentally: (1) aging hinders speech perception outcomes, or (2) aging affects the cross-modal plasticity responsible for an increase in the observed in the high group.

On the other hand, our linear regression model using only the preoperative amplitude of the P200 component at T8 accounted for 22% of speech perception variability after 6 months and up to 31% at 12 months of CI use. Given the relatively small sample size in our study, it is difficult to compare our predictions with those of large meta-analyses of audiometric data [34]. The preoperative classification proposed in our study might complement rather than replace multifactorial models based on clinical data, such that it might fill some of the 78% of unexplained variance left by clinical models. Future efforts that integrate both approaches might lead to more robust prediction of postoperative speech outcomes while shedding light on the mechanisms behind it. We emphasize the relevance of a preoperative marker that might help to predict which CI candidates could be at risk of poor postoperative speech outcome because this ability could improve preoperative counseling and inform the rehabilitation process.

For example, multisensory training might be of special benefit to these users [30,40]. There is an abundance of evidence of increased unisensory learning by multisensory training in humans and other animals [41,42,43]. Benefits of multi-over unisensory training include faster and more precise responses in a reduced number of training sessions [42]. Indeed, CI users who received audiovisual speech training, rather than auditory-only speech training, before and after implantation, had better postoperative speech perception; furthermore, good speechreading abilities correlated with good postoperative speech perception [15]. Therefore, future research could explore the use of coherent audiovisual speech training [41] in CI patients at risk of poor speech perception.

### 4.5. Study Design and Limitations

Before stating our conclusions, we would like to discuss some of the technical specifications and limitations of our study. Firstly, we chose to use visual evoked potentials because the high temporal resolution of EEG enables a more precise assessment of the contribution of the right temporal cortex to the different EEG components associated with the processing of basic stimuli features (P100, [18]), stimuli discrimination (N100, [19]) and attention allocation (P200, [20]). Secondly, we consider that the star-circle paradigm is well-suited to evaluate the recruitment of the temporal cortices for visual processing because it avoids speech content as a confounding factor [25]. This has been the case in a considerable amount of research that evaluated brain activity preoperatively during lipreading, the phonological judgment of written words, and audio–visual integration [5,14,15,30,36,38,44,45]. As reported by Fullerton and colleagues [25], activation of the temporal cortices in such paradigms corresponds to linguistic processing instead of recruitment for visual processing. Together, both research approaches provide a wider picture of the plasticity of the auditory cortices and speech perception in the context of postlingual hearing loss. Thirdly, the star-circle paradigm has been widely used in the field of cross-modal plasticity, and activation of the medial and the superior temporal gyri in response to this paradigm has been demonstrated using source localization analysis in high-density EEG [22] and further replicated [21,23]. So, despite the lower spatial resolution of EEG, activity at the temporal electrodes during EEG recordings during the star-circle paradigm reflects the activation of the temporal gyri. Another major limitation of our study was the small sample size which led to a decrease in statistical power after splitting the HL sample which allowed us to explore the interindividual variability and the potential for preoperative VEP components as predictors of postoperative speech perception outcome. However, replication studies in larger samples are essential to validate these results before incorporating this approach in clinical practice. Lastly, we still call for a cautious interpretation of our data in terms of spatial distribution, given the low spatial resolution of EEG in general, the lower electrode density used in our study compared to previous studies, and the analysis conducted at single electrode level instead of averages across different positions. However, we insist that low-resolution EEG (29 channels) is sufficient for the purpose of outcome prediction based on cross-modal plasticity and is more feasible to implement in everyday clinical screenings than PET, fMRI, or high-density EEG. Furthermore, using VEP can lead to high reproducibility across studies and subjects because, unlike phonological tasks, VEP does not rely on language.

## 5. Conclusions

Cross-modal plasticity provides additional information to audiometric measurements that might help predict postoperative speech perception outcomes after cochlear implant (CI) provision. It might also inform rehabilitation regimens to increase the likelihood of developing the best speech perception outcomes over time. In the present study, we showed that plasticity can have two directions in people with hearing loss (HL): larger amplitudes than normal hearing controls (NH) or smaller amplitudes than NH. These two plasticity profiles coincide with good or poor speech perception at 6 and 12 months of CI use. Postoperative speech perception outcomes correlated more strongly with preoperative cross-modal plasticity of the N100 and P200 components than with audiometric or with demographic factors, such as duration of hearing loss. Together, we consider that mixed models integrating audiometric and neurophysiological data hold great promise for understanding and predicting brain plasticity related to speech perception with cochlear implants. Still, the generalization of our results is limited by the relatively small sample size and further research is needed in order to understand (1) whether each component has a different susceptibility to be modified by cortical reorganization and (2) whether the adaptive or maladaptive effects of cross-modal plasticity are component-specific. In summary, integrating the assessment of cross-modal plasticity into preoperative evaluation might help to better predict postoperative outcomes and allow therapeutic training to be better adapted to the CI recipients’ needs, but further research is needed to establish the specific key biomarkers.

## Figures and Tables

**Table 1:** Demographic and audiometric data for both groups.

	Normal Hearing (*n* = 35)	Hearing Loss (*n* = 18)
Age (years)	62.10 (±7.48)	63.78 (±8.44)
Sex	M = 17, F = 18	M = 13, F = 5
4-PTA worse ear (dB)	19.30 (±8.62)	102.50 (±12.66)
4-PTA better ear (dB)	13.65 (±5.21)	80.21 (±13.21)

4-PTA: pure-tone average in dB (sound pressure level).

**Table 2:** Audiometric and demographic data of the for participants in the HL group.

Participant	Age (Years)	Sex	Duration of HL (Years)	CI Side	Freiburg Monosyllabic Speech Test (%)		
					PreOP	6 Mo CI Use	12 Mo CI Use
S1	65	M	15	R	0	60	90
S2	69	M	28	R	30	75	75
S3	58	M	8	L	0	65	60
S4	61	F	0.5	R	0	65	55
S5	45	M	2	R	0	80	75
S6	55	F	22	L	0	35	65
S7	62	M	12	L	0	50	75
S8	66	F	10	R	0	20	45
S9	77	M	8	R	0	70	70
S10	65	M	20	R	0	55	50
S11	63	M	24	R	0	40	NM
S12	64	M	19	L	40	NM	NM
S13	50	M	7	R	0	50	25
S14	63	M	20	R	0	NM	25
S15	68	F	10	L	0	50	30
S16	79	M	15	R	0	50	55
S17	65	F	30	R	5	50	80
S18	73	M	38	R	0	45	35

HL, Hearing loss; Mo, Months; NM, not measured.

**Table 3:** Spearman correlations between postoperative speech perception and preoperative demographic and audiometric data.

	df	6 Months CI Use	12 Months CI Use
Age (years)	14	−0.165 (0.541)	−0.041 (0.879)
Contralateral 4PTA	14	0.118 (0.663)	0.183 (0.498)
4PTA	14	0.202 (0.453)	0.351 (0.183)
Duration of HL	14	−0.413 (0.112)	0.114 (0.674)

Spearman rho (*p*-value). df, degrees of freedom (*n* − 2); 4PTA, 4-pure tone average (dB) prior to implantation; HL, hearing loss.

**Table 4:** Spearman correlations between postoperative speech perception and preoperative VEP latencies and amplitudes at T8.

	df	6 Months CI Use	12 Months CI Use
Latency of the P100 component at T8	13	−0.216 (0.439)	−0.079 (0.779)
Amplitude of the P100 component at T8	13	0.129 (0.646)	−0.149 (0.596)
Latency of the N100 component at T8	13	−0.258 (0.354)	0.065 (0.818)
Amplitude of the N100 component at T8	13	0.371 (0.173)	0.447 (0.095)
Latency of the P200 component at T8	13	−0.54 (0.038) *	−0.189 (0.499)
Amplitude of the P200 component at T8	13	−0.594 (0.019) *	−0.454 (0.089)

Spearman’s rho (*p* value). * *p* < 0.05. df, degrees of freedom. Statistically significant results are indicated in bold.

**Table 5:** Mean characteristics of each HL subgroup divided based on amplitude of the P200 component at T8.

	Low	High	Statistics		
			T_15_	*p*	Cohen’s d
Age (years)	66.0 ± 8.0	59.3 ± 7.8	−1.73	0.11	−0.85
4PTA	101 ± 13.2	106 ± 13.2	0.77	0.45	0.38
Contralateral 4PTA	80.3 ± 14.7	79.3 ± 13.8	−0.14	0.89	−0.07
Duration of HL	16.3 ± 7.6	12.5 ± 10.1	−0.89	0.39	−0.44

4PTA, 4-pure tone average in dB in the implanted ear prior to implantation; HL, hearing loss.

## Data Availability

Datasets reported in this publication are available upon reasonable request to the corresponding author.

## Appendix A

**Table A1:** Statistical comparisons by repeated measures ANOVA with Greenhouse–Geisser sphericity correction.

Factor		P100		N100		P200	
		Amplitude	Latency	Amplitude	Latency	Amplitude	Latency
Group	F	0.35	0.01	0.15	0.07	0.30	0.01
	df1	1	1	1	1	1	1
	df2	51	51	51	51	51	51
	p	0.558	0.974	0.698	0.786	0.585	0.909
Row	F	104.59	2.65	184.69	14.29	196.69	2.02
	df1	1.03	1.65	1.05	1.82	1.09	1.84
	df2	52.49	84.36	53.45	92.98	56.05	93.95
	p	**<0.001 *****	0.086	**<0.001 *****	**<0.001 *****	**< 0.001 *****	0.142
Row* group	F	0.31	1.29	0.08	0.28	0.15	0.22
	df1	1.03	1.65	1.05	1.82	1.09	1.84
	df2	52.49	84.36	53.45	92.98	56.05	93.95
	p	0.584	0.276	0.789	0.740	0.721	0.782
Laterality	F	10.85	0.21	7.71	0.79	12.61	3.11
	df1	1.70	1.61	1.91	1.70	1.70	1.95
	df2	86.86	82.15	97.50	87.08	87.04	99.58
	p	**<0.001 *****	0.762	**<0.001 *****	0.435	**<0.001 *****	0.05
Laterality*group	F	0.39	1.11	0.21	2.82	0.40	1.51
	df1	1.70	1.61	1.91	1.70	1.70	1.95
	df2	86.86	82.15	97.50	87.08	87.04	99.58
	p	0.643	0.324	0.805	0.073	0.637	0.226
Row* laterality	F	4.62	4.21	5.46	4.48	1.83	0.75
	df1	2.53	3.39	2.79	3.44	3.32	3.39
	df2	129.28	172.92	142.42	175.49	169.31	173.04
	p	**0.007 ****	**0.005 ****	**0.002 ****	**0.003 ****	0.138	0.537
Row* laterality* group	F	3.96	2.25	0.49	2.09	1.13	0.59
	df1	2.53	3.39	2.79	3.44	3.32	3.39
	df2	129.28	172.92	142.42	175.49	169.31	173.04
	p	**0.014 ***	0.076	0.677	0.093	0.340	0.641

Note. F and *p* values are shown for the three factors analyzed (Row: frontal, temporal, occipital; Laterality: left, temporal, right and Group: normal hearing (NH) and hearing impaired (HI)) df, degrees of freedom. Statistically significant results are indicated in bold. * *p* < 0.05, ** *p* < 0.01, *** *p* < 0.001.

**Table A2:** Latencies (ms) and amplitudes (µV) of VEP components at nine electrodes of interest.

	Latencies (ms)				Amplitudes (µV)			
Electrode	NH	HL	F	*p*	NH	HL	F	*p*
P100						P100		
F7	112 ± 10.4	114 ± 8.2	0.391	0.534	−3.26 ± 1.8	−4.13 ± 2.9	1.793	0.186
Fz	113 ± 12.7	113 ± 10.6	0.014	0.905	−3.85 ± 2.1	−4.19 ± 2.8	0.26	0.612
F8	111 ± 13.8	115 ± 9.8	0.988	0.325	−3.15 ± 1.8	−3.51 ± 2.9	0.305	0.583
T7	112 ± 10.7	109 ± 9.8	1.253	0.268	−2.16 ± 1.5	−1.90 ± 1.8	0.309	0.581
Cz	114 ± 11.5	111 ± 11.4	0.885	0.351	−2.99 ± 1.9	−3.32 ± 2.4	0.289	0.593
T8	109 ± 13.6	113 ± 8.9	1.054	0.309	−2.00 ± 1.6	−2.95 ± 3.8	1.583	0.214
O1	113 ± 10.6	112 ± 10.2	0.237	0.628	6.42 ± 5.0	5.91 ± 4.2	0.135	0.715
Oz	108 ± 13.0	108 ± 12.1	0.028	0.868	3.98 ± 3.8	4.33 ± 2.9	0.116	0.735
O2	112 ± 9.9	110 ± 11.1	0.447	0.507	5.09 ± 4.4	6.73 ± 6.4	1.208	0.277
N100						N100		
F7	164 ± 17.7	162 ± 19.0	0.160	0.691	2.96 ± 1.0	3.52 ± 1.6	2.370	0.130
Fz	162 ± 16.6	162 ± 19.3	0.008	0.929	3.30 ± 1.3	3.46 ± 1.7	0.142	0.708
F8	163 ± 19.5	165 ± 15.1	0.158	0.693	2.69 ± 1.0	2.83 ± 1.8	0.127	0.723
T7	162 ± 17.2	155 ± 16.7	1.949	0.169	2.26 ± 0.6	2.64 ± 1.5	1.771	0.189
Cz	161 ± 14.5	162 ± 16.0	0.106	0.746	2.95 ± 1.6	2.90 ± 2.0	0.009	0.923
T8	151 ± 21.5	160 ± 17.9	2.003	0.163	1.92 ± 0.9	2.06 ± 1.8	0.147	0.703
O1	158 ± 13.9	159 ± 11.9	0.067	0.797	−5.36 ± 3.5	−5.62 ± 4.5	0.054	0.817
Oz	151 ± 17.4	155 ± 13.6	0.759	0.388	−6.34 ± 3.8	−6.32 ± 4.9	0.000	0.992
O2	155 ± 16.6	156 ± 13.9	0.082	0.776	−6.30 ± 3.2	−6.46 ± 4.5	0.021	0.886
P200						P200		
F7	246 ± 17.7	241 ± 27.6	0.532	0.469	−1.81 ± 0.9	−2.25 ± 1.6	1.608	0.211
Fz	239 ± 25.6	243 ± 21.7	0.383	0.539	−1.61 ± 0.9	−1.76 ± 1.3	0.216	0.644
F8	245 ± 21.9	247 ± 16.6	0.099	0.754	−1.83 ± 0.6	−1.80 ± 1.6	0.008	0.930
T7	239 ± 18.9	234 ± 28.2	0.654	0.423	−1.69 ± 0.7	−1.99 ± 1.2	1.299	0.260
Cz	237 ± 23.3	241 ± 20.2	0.387	0.537	−1.25 ± 0.9	−1.18 ± 1.2	0.061	0.806
T8	241 ± 23.2	249 ± 15.9	1.537	0.221	−1.52 ± 0.7	−1.78 ± 1.3	0.976	0.328
O1	246 ± 23.6	245 ± 20.5	0.045	0.832	2.89 ± 1.6	3.08 ± 2.1	0.148	0.702
Oz	245 ± 23.2	243 ± 20.3	0.068	0.795	3.55 ± 1.8	3.65 ± 2.6	0.027	0.871
O2	247 ± 21.7	246 ± 20.3	0.011	0.918	3.48 ± 1.6	3.51 ± 2.5	0.003	0.959

NH, normal hearing; HL, hearing loss.
